# Airborne Endotoxin Concentrations in Homes Burning Biomass Fuel

**DOI:** 10.1289/ehp.0901605

**Published:** 2010-03-22

**Authors:** Sean Semple, Delan Devakumar, Duncan G. Fullerton, Peter S. Thorne, Nervana Metwali, Anthony Costello, Stephen B. Gordon, Dharma S. Manandhar, Jon G. Ayres

**Affiliations:** 1 Scottish Centre for Indoor Air, Population Health Sciences, University of Aberdeen, Aberdeen, United Kingdom; 2 Scottish Centre for Indoor Air, Institute of Occupational Medicine, Edinburgh, United Kingdom; 3 Centre for International Health and Development, University College London, London, United Kingdom; 4 Malawi-Liverpool-Wellcome Clinical Research Laboratories, Universities of Malawi and Liverpool (United Kingdom), Blantyre, Malawi; 5 Environmental Health Sciences Research Center, University of Iowa, Iowa City, Iowa, USA; 6 Mother and Infant Research Activities, Department of Paediatrics, Kathmandu Medical College, Kathmandu, Nepal; 7 Institute of Occupational and Environmental Medicine, University of Birmingham, Birmingham, United Kingdom

**Keywords:** biomass fuel smoke, endotoxin, inhalation, public health

## Abstract

**Background:**

About half of the world’s population is exposed to smoke from burning biomass fuels at home. The high airborne particulate levels in these homes and the health burden of exposure to this smoke are well described. Burning unprocessed biological material such as wood and dried animal dung may also produce high indoor endotoxin concentrations.

**Objective:**

In this study we measured airborne endotoxin levels in homes burning different biomass fuels.

**Methods:**

Air sampling was carried out in homes burning wood or dried animal dung in Nepal (*n* = 31) and wood, charcoal, or crop residues in Malawi (*n* = 38). Filters were analyzed for endotoxin content expressed as airborne endotoxin concentration and endotoxin per mass of airborne particulate.

**Results:**

Airborne endotoxin concentrations were high. Averaged over 24 hr in Malawian homes, median concentrations of total inhalable endotoxin were 24 endotoxin units (EU)/m^3^ in charcoal-burning homes and 40 EU/m^3^ in wood-burning homes. Short cooking-time samples collected in Nepal produced median values of 43 EU/m^3^ in wood-burning homes and 365 EU/m^3^ in dung-burning homes, suggesting increasing endotoxin levels with decreasing energy levels in unprocessed solid fuels.

**Conclusions:**

Airborne endotoxin concentrations in homes burning biomass fuels are orders of magnitude higher than those found in homes in developed countries where endotoxin exposure has been linked to respiratory illness in children. There is a need for work to identify the determinants of these high concentrations, interventions to reduce exposure, and health studies to examine the effects of these sustained, near-occupational levels of exposure experienced from early life.

The use of solid or biomass fuels to cook and to heat homes is widespread in large parts of the developing world, with an estimated 3 billion people exposed to smoke from burning these fuels in their own home (International Energy Agency and Organisation for Economic Co-operation and Development 2004). The World Health Organization estimates that biomass fuel smoke exposure is responsible for about 1.5 million early deaths per year (Prüss-Ustün et al. 2008), with a global burden of disease of approximately 2.5% of all healthy life-years lost. Most of the burden of disease arises from respiratory infections, especially in children < 5 years of age, with a disproportionate amount of health problems falling on women and children, who are more likely to be at home or to have responsibilities for cooking and heating activities (Rehfuess et al. 2006).

Research into indoor air pollution in homes burning biomass fuels has tended to focus on airborne concentrations of fine particulate matter (PM) (Albalak et al. 2001; Edwards et al. 2007; Ezzati et al. 2000; Fullerton et al. 2009; Kurmi et al. 2008), but airborne endotoxin may also play an important role.

Endotoxin or lipopolysaccharide is part of the cell wall of Gram-negative bacteria and has been measured in airborne PM in occupational settings, office buildings, households, and ambient air. Once inhaled, endotoxin stimulates an amplifying series of endotoxin–protein and protein–protein interactions, sequentially binding to a range of proteins and receptors, leading to production of chemotactic cytokines and chemokines (Hađina et al. 2008) and lung inflammation and resultant oxidative stress. Studies have shown associations between household endotoxin concentrations and diagnosed asthma, asthma medication use, and severity of asthma symptoms (Michel et al. 1996; Thorne et al. 2005). Respiratory illness in endotoxin-exposed working populations has been frequently documented (Smit et al. 2008; Thorne and Duchaine 2007). In asthma, endotoxin exposure has been shown to be protective of development of allergic disease at low levels while also producing nonallergic asthma and/or aggravating symptoms of existing asthma (Douwes et al. 2003; Smit et al. 2009).

Thorne and Duchaine (2007) tabulated data on endotoxin levels in a wide variety of industries and home environments, which indicated geometric mean (GM), inhalable fraction, personal exposures of 12–8,300 endotoxin units (EU)/m^3^ in agricultural occupations and 5.8 EU/m^3^ in homes of rural asthmatic children (*n* = 326).

Airborne endotoxin concentrations in homes in Boston, Massachusetts (USA), have been shown to be significantly associated with the presence of dogs, moisture sources in the home, and the amount of settled dust (Park et al. 2001). Endotoxin has also been identified in tobacco smoke (Larsson et al. 2004) and in homes where smoking takes place, pets are present, and/or dampness or mold is found (Rennie et al. 2008; Tavernier et al. 2006). In the large U.S. National Survey of Endotoxin in Housing (Vojta et al. 2002), increased household endotoxin was most strongly associated with living in poverty, number of people in the home, pet ownership, and household cleanliness (Thorne et al. 2009). Most studies have used endotoxin levels in settled dust as a surrogate for personal exposure. There are very few studies that have measured airborne endotoxin concentrations in household settings.

It seems probable that the burning of common biomass fuels such as wood, charcoal, dried animal dung, and crop residues within small and poorly ventilated homes will produce high endotoxin exposures. The only available report in the scientific literature comes from a small study in the Ladakh region of India, where short-term sampling (< 60 min) of two homes produced average endotoxin concentrations of 24 and 190 EU/m^3^ (Rosati et al. 2005). These concentrations are within the range of those found in occupations involved in the handling and processing of large volumes of biological material.

In this article we present results from a study to measure endotoxin levels within the main living area of 69 homes in Malawi and Nepal and to explore differences in these concentrations based on the fuel type being used.

## Materials and Methods

### Study population and sampling strategy

Samples of airborne PM were collected from homes in two studies that assessed indoor air pollution and health in Malawi and Nepal. In Nepal the Dhanusha district was selected. This is a flat, low-lying area of the country close to the border with India. Two villages were sampled: one in the south of the district (Lohana), where dried cow or buffalo dung is burned, and one in the north (Dhalkebar), where wood is burned. Fifteen homes were sampled in each village, during cooking time in the morning or evening in December 2008. After consent was given by an adult householder, air sampling equipment was placed in the main living area of the home and sampled air between 90 and 180 min. This study had ethical approval from the Nepal Health Research Council.

For the Malawi study, details of methods used and results of PM concentrations measured have been previously published (Fullerton et al. 2009). In summary, a total of 75 homes were recruited from around Blantyre and rural Chikwawa villages during April 2008. Sampling equipment was placed in the main living area of each of these homes for a period of approximately 24 hr, except in six homes where short-term sampling similar to that used in Nepal (60–200 min duration around the time of a cooking event) was carried out (all respirable samples; *n* = 4 wood burning; *n* = 2 maize crop residue burning). Not all homes received an instrument capable of providing a sample for the measurement of endotoxin concentrations; we therefore present a subsample of data from 38 (19 rural and 19 urban) of the 75 Malawian homes. This study had ethical approval from the Research Ethics Committee of the College of Medicine, University of Malawi, and the Liverpool School of Tropical Medicine.

### Sample collection

Air sampling was conducted by placing a small Apex air pump (Casella, Bedford, UK) attached to either a cyclone sampling head (2.2 L/min) or an Institute of Occupational Medicine sampling head (2.0 L/min) to sample the respirable (median aerodynamic diameter, 4 μm) or the total inhalable (defined as anything that can be breathed into the nose and mouth and is broadly particulate with an aerodynamic diameter < 100 μm) particle size fraction of PM, respectively. Both types of sampling heads were loaded with preweighed 25-mm glass-fiber filters with a 0.7-μm pore size. All samples in Nepal were collected using an IOM sampling head, whereas 32 of the 38 Malawian samples were collected using a cyclone. Sampling was performed in accordance with Methods for Determination of Hazardous Substances (MDHS) no. 14/3 (Health and Safety Executive 2000). The equipment was placed in the main living area of the home at a height of approximately 1.0 m and, where possible, at about 1.0 m from the main stove or cooking area. After sampling, each filter was placed in a sealed metal tin and sent back to the United Kingdom for reweighing before being further transported to the United States for endotoxin analysis. Field blanks were used to correct the data for changes in filter weight associated with manipulation.

### Endotoxin analysis

Endotoxin concentrations of samples were measured using a modification of the kinetic chromogenic *Limulus* amebocyte lysate assay (Lonza, Inc., Walkersville, MD, USA) (Thorne et al. 2005). Briefly, air sampling filters were extracted in sterile, pyrogen-free water containing 0.05% Tween 20 for 1 hr at 22°C, with continuous shaking. Filter extracts were centrifuged 20 min at 600 × *g*. Two-fold serial dilutions of endotoxin standards (*Escherichia coli* O111:B4) and 5-fold serial dilutions of sample extracts were prepared using sterile, pyrogen-free water in heat-treated borosilicate glass tubes. A 13-point standard curve was generated ranging from 0.025 to 100 EU/mL (*R*^2^ > 0.995), with absorbance measured at 405 nm (SpectraMax 340; Molecular Devices, Inc., Sunnyvale, CA, USA). Endotoxin determinations were based upon the maximum slope of the absorbance versus time plot for each well.

The arithmetic mean (14.4 EU/sample) for the six Malawi filter field blanks was subtracted from each of the other Malawi filter results. The analytical limit of detection (LOD) was derived from using a value of three times the standard deviation (9.24 EU/filter) of the field blank measurements (Malawi filter analytical LOD = 27.7 EU/filter). Where corrected filter values were less than the LOD (*n* = 12), the filter was assigned a value of one-half the LOD (13.9 EU/filter). A similar process was applied to the Nepal filters based on results from four field blanks (arithmetic mean = 4.4; SD = 0.95 EU/filter; analytical LOD = 2.85 EU/filter). For the Nepal filters with corrected values less than the LOD (*n* = 4), a value of 1.43 EU/filter was assigned.

### Statistical analysis

Data were double entered to a Statistical Package for the Social Sciences (SPSS), version 17.0 file (SPSS Inc., Chicago, IL, USA), and summary statistics and box plots were generated directly. Mean endotoxin concentrations measured in Nepalese total inhalable dust samples from wood- and dung-burning homes were compared using a Mann–Whitney *U*-test. A similar test was used to test for differences in respirable endotoxin concentrations in Malawian charcoal- and wood-burning homes.

## Results

Tables 1 and 2 provide summary statistics of the measured total inhalable and respirable dust concentrations and the endotoxin concentrations measured in the homes. Data are subdivided by country, primary fuel type of the home, and measurement duration. The PM concentrations from the short-duration samples (Table 1) are generally about an order of magnitude higher than the 24-hr samples collected in Malawi, reflecting the much higher smoke concentrations during cooking events than at other times in the household. Total inhalable endotoxin concentrations during cooking-time sampling show much higher median values during dung burning in Nepal (365 EU/m^3^) than during wood burning in Nepal (43 EU/m^3^). For 24-hr samples, total inhalable endotoxin median values were higher in wood-burning (40 EU/m^3^) than in charcoal-burning (24 EU/m^3^) homes. Although values for respirable endotoxin concentrations are not directly comparable with total inhalable endotoxin concentrations and will be an underestimate of total inhalable levels, the respirable data are broadly supportive of the increasing gradient in endotoxin concentrations: charcoal < wood < cow dung < maize crop residues.

**Figure 1:**
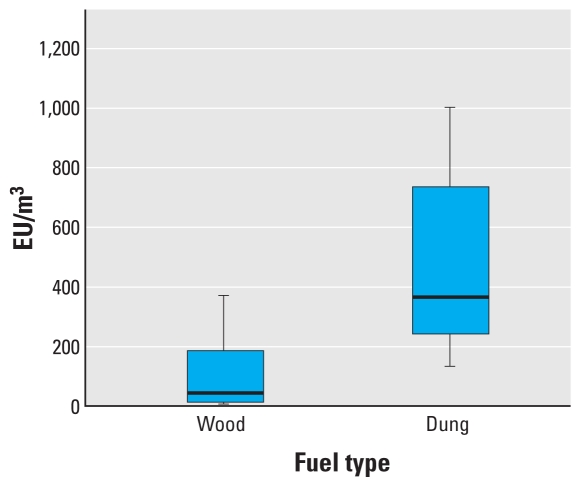
Box plot of airborne total inhalable endotoxin concentrations by fuel type during cooking in Nepalese homes. The line inside the box represents the median value, the lower and upper box lines represent the limits of the interquartile range (25th and 75th percentiles), and the “whiskers” represent the 5th and 95th percentiles of the distribution. Difference in means *p* < 0.01.

**Figure 2:**
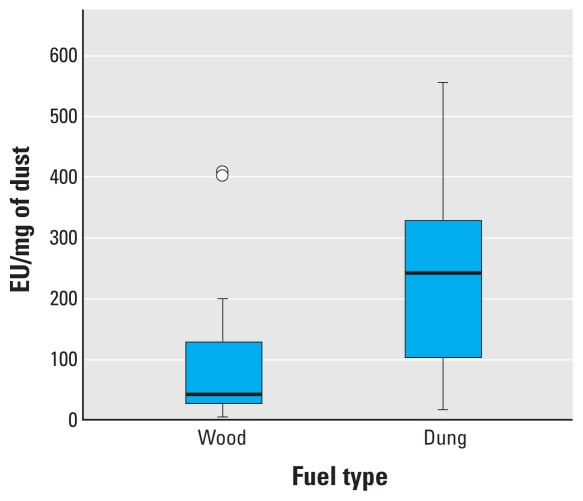
Box plot of airborne endotoxin by fuel type during cooking per PM mass on the filter in Nepalese homes. The line inside the box represents the median value, the lower and upper box lines represent the limits of the interquartile range (25th and 75th percentiles), and the “whiskers” represent the 5th and 95th percentiles of the distribution. Circles indicate outlier observations with values 1.5–3.0 times the interquartile range from the 25th or 75th percentile. Difference in means *p* = 0.024.

Figure 1 is a box plot of airborne endotoxin concentrations from the directly comparable samples taken during cooking from wood-burning and dung-burning homes in Nepal. There is a statistically significant difference in airborne endotoxin concentrations in Nepalese homes burning dung compared with those burning wood (Mann–Whitney *U*-test, *z* = 4.0; *p* < 0.01). The much larger endotoxin concentrations in dung-burning homes do not appear to be simply a function of the increased PM produced in this type of fuel. Figure 2 illustrates the amount of endotoxin per mass of PM measured in the Nepalese villages and demonstrates that dung-generated smoke tends to contain much more endotoxin than does a similar mass of wood-generated smoke (*z* = 2.2; *p* = 0.024).

**Figure 3:**
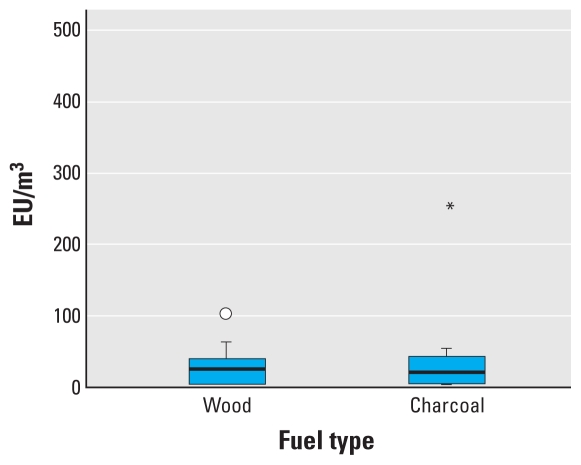
Box plot of 24-hr airborne respirable endotoxin concentrations by fuel type in Malawian homes. The line inside the box represents the median value, the lower and upper box lines represent the limits of the interquartile range (25th and 75th percentiles), and the “whiskers” represent the 5th and 95th percentiles of the distribution. The circle indicates an outlier observation as described in Figure 2; the asterisk indicates an observation more than three times the interquartile range from the 25th or 75th percentile. Difference in means *p* = 0.647.

**Figure 4:**
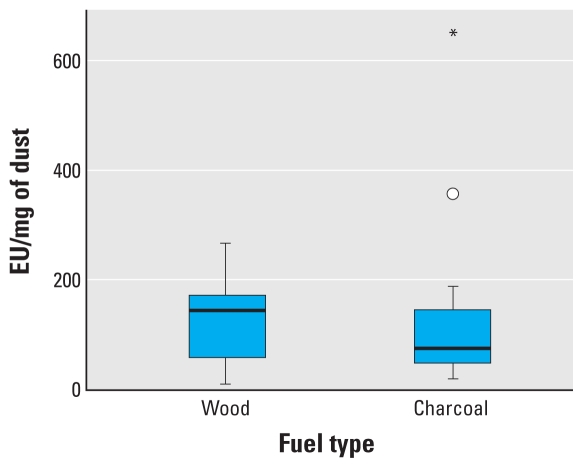
Box plot of 24-hr airborne respirable endotoxin by fuel type per PM mass on the filter in Malawian homes. The line inside the box represents the median value, the lower and upper box lines represent the limits of the interquartile range (25th and 75th percentiles), and the “whiskers” represent the 5th and 95th percentiles of the distribution. The circle indicates an outlier observation as described in Figure 2; the asterisk indicates an observation more than three times the interquartile range from the 25th or 75th percentile. Difference in means *p* = 0.808.

Figure 3 presents data on 24-hr respirable endotoxin concentrations from the Malawian homes burning charcoal or wood. The difference between fuel types is not statistically significant (*z* = 0.46; *p* = 0.647). Figure 4 shows the median endotoxin concentration per mass of respirable PM, again for the 24-hr samples collected in homes in Malawi. The median concentrations of endotoxin per mass of dust is higher in wood-burning homes than in charcoal-burning homes, although this is not statistically significant (*z* = 0.243; *p* = 0.808).

## Discussion

Endotoxin concentrations reported in this study are high and much higher than those found in a recent study measuring airborne endotoxin in 10 homes in northern California (Chen and Hildemann 2009), where mean concentrations were generally < 1 EU/m^3^, and in a study of homes of rural asthmatic children, where the GM inhalable endotoxin was 5.8 EU/m^3^ (*n* = 326) (Thorne and Duchaine 2007). They were also considerably higher than those measured from a large study of the homes of 332 children in Canada (Dales et al. 2006). The mean airborne endotoxin concentration in the Canadian study was 0.49 EU/m^3^, almost 100 times less than the 24-hr average levels measured in this study for charcoal-burning homes and close to 1,000 times lower than the average level during cooking with dried dung in homes in Nepal. However, results from the Canadian study showed that even at the relatively low levels of exposure experienced by the Canadian study population, there was a statistically significant relationship between airborne endotoxin and respiratory illness in the first 2 years of life.

The only previous study of endotoxin concentrations in biomass-burning homes was carried out in two homes in the Ladakh region of India (Rosati et al. 2005), where endotoxin levels of 24 and 190 EU/m^3^ were found, broadly in line with our data. The Indian homes were small, portable tentlike structures with little in the way of ventilation or extraction of smoke generated from burning dung and crop residues.

A health-based guidance limit of 50 EU/m^3^ has been recommended for occupational settings in the Netherlands (Heederik and Douwes 1997) for an 8-hr time-weighted average exposure. The median value of 24-hr samples collected from charcoal-burning homes (using respirable dust size selection and hence conservative compared with the total inhalable dust sampler used for the limits proposed in the Netherlands) was approximately 20 EU/m^3^. Scaling this to an 8-hr time-weighted average would produce levels of around 60 EU/m^3^, exceeding the concentration deemed to be acceptable for a healthy workforce. From our results, we would anticipate much higher 8-hr time-weighted average values from wood- and dung-burning homes, and it seems likely that many of these would approach or exceed the health-based guidance limit value.

The health effects of exposure to the endotoxin concentrations measured in the homes in this study may be considerable, particularly because exposure is sustained and occurs from birth in most homes. Personal exposures of women who carry out cooking and fire lighting have the potential to be even higher than the static or area measurements made in this study because of regular close proximity to the smoke plume. There is a need for personal exposure data in these settings.

We acknowledge that this study has several important weaknesses. We did not design the study to collect samples for analysis of endotoxin, but rather “piggy-backed” it onto two studies that set out to characterize PM concentrations in homes in Malawi and Nepal. As a consequence, our results present data from both short cooking periods and longer 24-hr samples and also a mixture of total inhalable and respirable PM size selection. In addition, there was an extended period between the collection of the filters and analysis for endotoxin, and we believe that this led to the high levels of contamination of some of the field blanks that we have reported. This is particularly evident for the Malawi samples, which were stored for the longest duration. We report our data separately by size fraction, sampling duration, country, and fuel type and used appropriate methods for blank correction to overcome these weaknesses where possible.

Further work should use a standard protocol for endotoxin measurement and should seek to standardize durations of sample collection. Optimally, personal exposure measurements should be considered, especially in the context of health-related exposure measurement. Our study design collected only two samples from homes burning crop residues, and any future study should seek to address this data gap.

Controlling and reducing exposure to biomass fuel smoke in homes in the developing world are complex and difficult areas with such options as modifications of behavior, introduction of better and more efficient stoves, and improved household ventilation (Zhang and Smith 2007). Methods of reducing airborne endotoxin concentrations will be broadly similar, but there may also be opportunities to reduce bacterial and endotoxin content of the source fuel via harvesting and/or production methods and changes to how fuel is stored. Higher cooking temperatures are likely to degrade endotoxin, and more efficient cooking using improved stove technologies can also reduce the generation of PM-bound endotoxin. A recent study has also suggested that outdoor storage of wood chips increased endotoxin content (Sebastian et al. 2006), so dry, indoor storage areas for fuel may reduce the airborne endotoxin levels when burning eventually takes place.

Our study raises the possibility of an important new risk factor, and preventive strategies, for respiratory morbidity and mortality in the developing world. The mechanism for the association between biomass smoke exposure and infections of the lower respiratory tract in children remains unclear but is likely to be multifactorial and influenced by housing conditions, nutritional status, and other coexposures. It is possible that inhaled endotoxin, being proinflammatory, may be one contributory factor in this mechanistic pathway. Pneumonia remains one of the largest contributors to under-five mortality, and exposure to high concentrations of airborne endotoxin may be an important risk factor for the severity of illness (Dales et al. 2006). From a public health perspective, interventions to reduce PM and endotoxin exposures generated from household combustion of solid fuels should be implemented as a matter of urgency.

## Conclusions

This study has shown that airborne endotoxin concentrations in homes burning biomass fuels are considerably higher than those found in homes in the developed world and at levels comparable to agricultural-related occupations. Some homes recorded cooking period concentrations > 1,000 EU/m^3^, more than 20 times the health-based occupational guidance limit suggested in the Netherlands. There is a need for a larger study using a standard protocol that allows further identification of the determinants of exposure in these homes. This would increase our understanding of which fuels produce the high levels. Methods to separate the influence of endotoxin concentrations from those of high airborne PM levels are also required, as are epidemiologic and intervention studies to determine the health effects of reducing exposure to these high endotoxin levels.

## Figures and Tables

**Table 1:** Summary statistics of PM and endotoxin concentrations by fuel type, sampling fraction, and country for short (< 200 min) cooking-interval samples.

	PM (mg/m^3^)				Endotoxin (EU/m^3^)			
Fuel/particle size	*n*	Range	Mean ± SD	Median	Range	Mean ± SD	Median	< LOD (*n*)
Nepal, TIP								
Wood	16	0.22–4.08	1.14 (1.12)	0.68	6–371	100 (113)	43	4^a^
Dung	15	0.91–12.8	3.78 (3.51)	2.33	133–1,002	498 (291)	365	0

Malawi, Resp								
Wood	4	1.40–9.65	4.73 (3.97)	3.94	63–520	202 (217)	113	1^b^
Maize crop residue	2	0.65–9.56	5.11 (6.30)	5.11	45–3,172	1,609 (2,211)	1,609	1^c^

Abbreviations: LOD, analytical limit of detection (Malawi, 27.7 EU/filter; Nepal, 2.85 EU/filter); Resp, respirable PM; TIP, total inhalable PM.

a Concentrations generated from filters assigned values of LOD/2 were 6.18, 6.23, 7.02, and 7.96 EU/m^3^.

b Concentration generated from filter assigned values of LOD/2 was 63.3 EU/m^3^.

c Concentration generated from filter assigned values of LOD/2 was 45.1 EU/m^3^.

**Table 2:** Summary statistics of PM and endotoxin concentrations by fuel type and sampling fraction for 24-hr Malawi samples.

	PM (mg/m^3^)				Endotoxin (EU/m^3^)			
Fuel/particle size	*n*	Range	Mean ± SD	Median	Range	Mean ± SD	Median	< LOD
Wood								
TIP	4	0.43–0.81	0.65 (0.18)	0.68	34–141	64 (52)	40	0
Resp	9	0.03–0.70	0.32 (0.25)	0.24	5–106	31 (34)	26	4^a^

Charcoal								
TIP	2	0.20–0.32	0.26 (0.09)	0.26	21–26	24 (3.6)	24	0
Resp	17	0.04–0.72	0.25 (0.17)	0.23	4–256	35 (59)	21	6^b^

Abbreviations: LOD, analytical limit of detection (Malawi, 27.7 EU/filter; Nepal, 2.85 EU/filter); Resp, respirable PM; TIP, total inhalable PM.

a Concentrations generated from filters assigned values of LOD/2 were 4.88, 5.22, 5.24, and 5.40 EU/m^3^.

b Concentrations generated from filters assigned values of LOD/2 were 4.08, 4.26, 4.29, 4.60, 5.20, and 5.31 EU/m^3^.
